# Relationship between Lung Carcinogenesis and Chronic Inflammation in Rodents

**DOI:** 10.3390/cancers13122910

**Published:** 2021-06-10

**Authors:** Yuko Nakano-Narusawa, Masanao Yokohira, Keiko Yamakawa, Juanjuan Ye, Misa Tanimoto, Linxuan Wu, Yuri Mukai, Katsumi Imaida, Yoko Matsuda

**Affiliations:** Oncology Pathology, Department of Pathology and Host-Defense, Faculty of Medicine, Kagawa University, 1750-1 Ikenobe, Miki-cho, Kita-gun, Kagawa 761-0793, Japan; y_nakano@med.kagawa-u.ac.jp (Y.N.-N.); yokohira@med.kagawa-u.ac.jp (M.Y.); yamakawa@med.kagawa-u.ac.jp (K.Y.); jj528575963@gmail.com (J.Y.); mi-tanimoto@med.kagawa-u.ac.jp (M.T.); s20d702@stu.kagawa-u.ac.jp (L.W.); mky-39@med.kagawa-u.ac.jp (Y.M.); imaida.katsumi@kagawa-u.ac.jp (K.I.)

**Keywords:** lung carcinogenesis, tobacco related substances, chronic inflammation, rodent model

## Abstract

**Simple Summary:**

Lung cancer is the most common cause of cancer-related deaths worldwide. There are various risk factors for lung cancer, including tobacco smoking, inhalation of dust particles, chronic inflammation, and genetic factors. Chronic inflammation has been considered a key factor that promotes tumor progression via production of cytokines, chemokines, cytotoxic mediators, and reactive oxygen species by inflammatory cells. Here, we review rodent models of lung tumor induced by tobacco, tobacco-related products, and pro-inflammatory materials as well as genetic modifications, and discuss the relationship between chronic inflammation and lung tumor. Through this review, we hope to clarify the effects of chronic inflammation on lung carcinogenesis and help develop new treatments for lung cancer.

**Abstract:**

Lung cancer remains the leading cause of cancer-related deaths, with an estimated 1.76 million deaths reported in 2018. Numerous studies have focused on the prevention and treatment of lung cancer using rodent models. Various chemicals, including tobacco-derived agents induce lung cancer and pre-cancerous lesions in rodents. In recent years, transgenic engineered rodents, in particular, those generated with a focus on the well-known gene mutations in human lung cancer (*KRAS*, *EGFR*, and *p53* mutations) have been widely studied. Animal studies have revealed that chronic inflammation significantly enhances lung carcinogenesis, and inhibition of inflammation suppresses cancer progression. Moreover, the reduction in tumor size by suppression of inflammation in animal experiments suggests that chronic inflammation influences the promotion of tumorigenesis. Here, we review rodent lung tumor models induced by various chemical carcinogens, including tobacco-related carcinogens, and transgenics, and discuss the roles of chronic inflammation in lung carcinogenesis.

## 1. Introduction

Cancer is the first or second leading cause of death before the age of 70 years in numerous countries cause of death worldwide, and was responsible for an estimated 10.0 million deaths in 2020 [1]. An estimated 19.3 million new cancer cases and almost 10.0 million cancer deaths occurred in 2020 [2]. Lung cancer remained the leading cause of cancer death, with an estimated 1.8 million deaths [1]. Approximately 80–90% of lung cancer cases are associated with the carcinogens released during the combustion of tobacco, either as first- or second-hand smoke. International variation in lung cancer rates and trends largely reflects the maturity of the tobacco epidemic [3]. Other than tobacco, there are various risk factors, such as radon, inhalation of fine or nano particles (e.g., asbestos, clistalin silica, etc.), domestic fuel smoke (biomass), e-cigarettes, occupational hazards, air pollution, sex, steroids, infectious diseases, interstitial pneumonia (especially idiopathic pulmonary fibrosis), previous radiation therapy of the lungs and family history of lung cancer [4,5,6,7,8]. Although genetic mutations underlie malignant transformation, the presence of mutations alone is not sufficient for tumor formation, and additional alterations are necessary for the development of cancer [9,10]. Carcinogenesis is a complex, stepwise process that involves the acquisition of genetic mutations and epigenetic changes that alter cellular processes, such as proliferation, differentiation, invasion, and metastasis [11]. In addition to the environmental and hereditary factors, stochastic effects, which are random mutations arising during DNA replication in non-cancerous stem cells, are involved in the development of cancer [12]. Among the multiple factors initiating and supporting tumor growth, inflammation plays one of the most important roles [13,14].

Inflammation is a tissue response to a variety of harmful stimuli, such as pathogens, irritants, and injuries, and can eliminate tissue damage. However, dysregulated inflammation is a recognized cause of many human diseases, exemplified by organ fibrosis and cancer [15]. Etiologic studies revealed close relationship between chronic inflammation and lung cancer induced by inhalation of dust particles, such as silica and asbestos. Furthermore, we reported that quartz or the other particles enhanced inflammation and tumor formation in rodent lungs [13,16,17,18,19,20,21,22,23]. In this review, we discuss the relationship between lung carcinogenesis and chronic inflammation in rodent models.

## 2. Rodent Models of Lung Tumorigenesis

### 2.1. Rodent Lung Tumor Models of Tobacco Smoke and Smoking-Related Substance Carcinogenesis

The geographic and temporal patterns of lung cancer incidence as well as mortality at a population level are mainly determined by tobacco consumption, the main etiological factor of lung carcinogenesis. Approximately 75% of lung cancer cases in the world are attributable to tobacco smoking [24]. More than 55 out of the 4000 chemicals in tobacco smoke have carcinogenic activity according to the International Agency for Research on Cancer (IARC), an intergovernmental agency that is a part of the World Health Organization of the United Nations [25,26].

**Figure 1:**
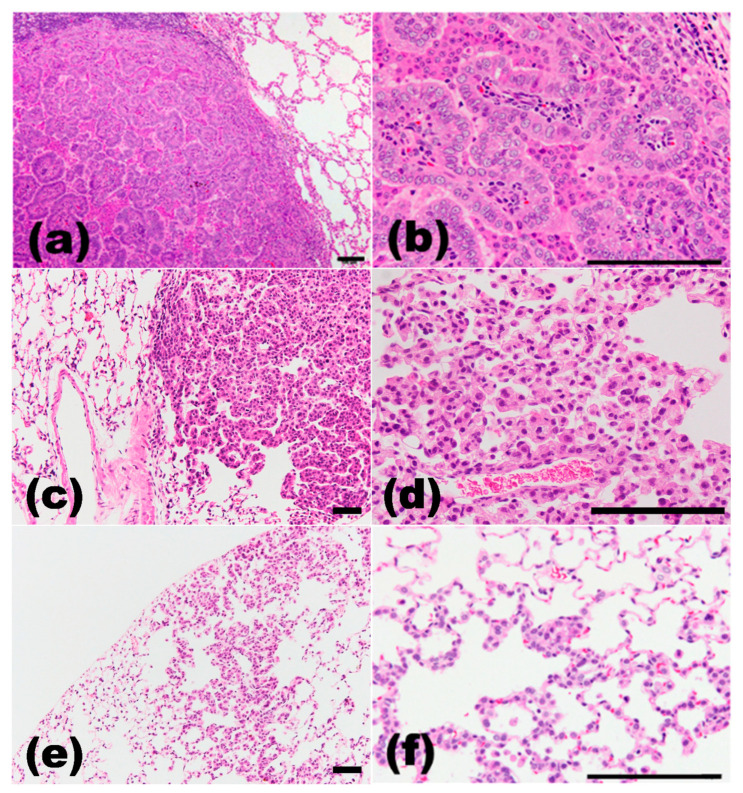
Histopathological findings in DHPN-induced rat lung proliferative lesions. (**a**,**b**), adenocarcinoma; (**c**,**d**), adenoma; (**e**,**f**), hyperplasia. (**a**,**c**,**e**), low magnification; (**b**,**d**,**f**), high magnification. H&E staining. Scale bar, 100 μm.

Although nicotine induces addiction in humans, nicotine itself is not considered carcinogenic. However, we reported that nicotine promoted neurologic symptoms during the acute phase, and strong inflammation, e.g., neutrophil infiltration, edema and fibrosis, in the lungs during the chronic phase, even at a low dose. Histopathologically, though proliferative changes were not observed [27]. Cigarette contains a mixture of carcinogens, including a small dose of polycyclic aromatic hydrocarbons (PAHs) and 4-(methylnitrosamino)-1-(3-pyridyl)-1-butanone (NNK), that are tumor promoters and co-carcinogens in the lungs [28]. Tobacco smoke includes many carcinogens, such as PAH, N-Nitrosamines including NNK, aza-arenes, aromatic amines, heterocyclic amines, aldehydes, miscellaneous organic compounds, and inorganic compounds [28]. N-nitrosodiethylamine (DEN) an N-Nitrosoamine [29,30,31], and benzo[a]pyrene (BaP), a PAH [32,33], are well-known carcinogens in tobacco smoke and induce carcinoma in almost all human tissues, including the lungs, mouth, throat, larynx, esophagus, stomach, colon, kidneys, liver, pancreas, cervix, bladder, and blood. However, tobacco smoke inhalation assays in laboratory animals show low incidence of pulmonary tumor formation, and most of the tumors are adenomas with the occasional adenocarcinoma as opposed to the highly invasive squamous cell carcinoma seen in human smokers [34]. NNK is considered a strong carcinogen for tobacco-related lung cancer in humans and rodents [35]. In our study, the A/J mouse models using NNK induced development of lung tumors, including hyperplasias, adenomas, and adenocarcinomas (Figure 1). Oral administration or peritoneal injection of NNK induced adenoma and adenocarcinoma in the lungs of both sensitive and resistant strains of mice [28], while exhibiting low carcinogenic effects in other organs. In addition, small cell or squamous cell carcinomas were rarely seen in these models. Urethane, another natural constituent of tobacco, is also present in tobacco smoke and causes malignant lung tumors [36]. Urethane, administered through drinking water, induced lung inflammation, alveolar and bronchiolar hyperplasia, alveolar/bronchiolar adenomas, nephropathy, cardiomyopathy, lymphoid and bone marrow cell depletion, seminiferous tubule degeneration, and ovarian atrophy and follicular degeneration in mice [37]. In male Syrian golden hamsters were treated with DEN with or without exposure to tobacco smoke to investigate the potential short-term promoting effects of cigarette smoke on the development of tumors in the respiratory system. Bronchial epithelial hyperplasia and squamous papilloma were observed in DEN-treated lungs and small aggregations of macrophages in the smoke-exposed lung alveoli [38]. In addition, exposure to tobacco smoke induces pulmonary adenocarcinomas in murine models and establishment of such models allows the testing of potential chemopreventive strategies [39,40].

Genetic susceptibility, occupational exposures, air pollution, and eating habits (high intake of meat, in particular fried or well-done red meat, coffee and alcohol consumption) may act independently or in concert with tobacco smoking in development of lung cancer [41]. It has been reported that women are more susceptible to tobacco smoke and have a higher incidence of tobacco-related lung cancer than men [42]. Lung cancers in the never-smokers is more common in women, especially Asian women, than men [43]. Previously, we reported the correlations between tumor size and epidermal growth factor receptor (EGFR) expression, as well as between EGFR and progesterone receptor expression in NNK-induced female mouse lung adenocarcinomas. Notably, adenoma and adenocarcinoma were significantly suppressed by ovariectomy [44,45]. Further studies are needed to clarify the roles of sex hormones and race on lung cancer.

### 2.2. Carcinogens Used in Rodent Lung Tumor Models

Table 1 shows the main chemical-induced lung carcinogenesis rodent models, and most of them were related to tobacco smoke exposure. N-bis(2-hydroxypropyl)nitrosamine (DHPN), a nitrosamine, is a dipropylamine in which the hydrogen attached to the nitrogen is replaced by a nitroso group, and the synthesized carcinogen does not exist in nature [46,47]. It is a carcinogen targeting the lungs, liver, thyroid gland, kidneys, etc. [13,17,19,20,22,48,49,50]. DHPN induced double-strand breaks in pulmonary epithelial cells, as determined by the expression of γH2AX that is formed as a result of a double-strand break and can act as a sensitive marker of genomic instability [51,52]. In our study, the rodent tumor model generated using DHPN induced lung tumor, including hyperplasias, adenomas, and adenocarcinomas (Figure 1). DHPN also induced squamous cell carcinomas at low incidence, and most of them were adenocarcinomas with squamous cell metaplasia [13] (Table 1).

Aflatoxin B_1_ is a mycotoxin and a member of a family of difuranocoumarins produced by *Aspergillus flavus* and related fungi. The lungs and liver are targets of aflatoxin B_1_, following dietary or inhalational exposure [53,54]. Oral administration of aflatoxin G_1_, another member of the carcinogenic aflatoxin family, caused tumor necrosis factor (TNF)-α-dependent inflammation that enhanced oxidative DNA damage in alveolar epithelial cells, which in turn may be related to aflatoxin G_1_-induced lung carcinogenesis [55].

Similarly, 4-nitroquinoline 1-oxide (4-NQO) is a pluripotent carcinogen in several tissues, and is frequently used to induce oral and lung cancers in vivo [56,57]. It is thought to elicit its carcinogenicity by producing DNA adducts after being metabolized to 4-hydroxyaminoquinoline 1-oxide, which forms 8-hydroxydeoxyguanosine (8OHdG), a marker of oxidative damage [58].

Several rodent models of lung adenocarcinoma have been reported; however, there are few reports on rodent models of other types of lung cancers, including squamous cell carcinomas that is the most common human lung cancer type. N-nitroso-tris-chloroethylurea (NTCU), one of components of nitrosoalkylureas, induced squamous cell carcinomas in mice at high incidence [59,60]. Furthermore, intravenous injection of H69/VP cells (human small cell carcinoma cell line) into SCID mice formed lung small cell carcinoma with liver and systemic lymph node metastases [61].

### 2.3. Gene Mutation and Transgenic Animal Models

Kirsten rat sarcoma viral oncogene homolog (*KRAS*) and *EGFR* are the most frequently mutated oncogenes in human lung adenocarcinoma [82,83]. *KRAS* mutations are associated with a strong history of tobacco smoking, whereas *EGFR* mutations are the most frequent oncogene alterations in lung tumors of never-smokers [24,83]. Gene mutations, such as *KRAS* and *EGFR*, have been studied in various chemical carcinogen-induced rodent lung tumors. In rodent lung carcinogenesis models, *KRAS* mutations are frequently detected, whereas genetic alteration in *EGFR* is rare. A silent mutation in *EGFR* exon 20 (AAAAC→AA T; N772) was detected in DHPN-induced rat lung tumor. Activating mutations in *KRAS* codon 12, G/C→A/T transitions, were commonly detected in NNK-induced mouse tumors (5/6, 83%), MeIQx-induced mouse tumors (1/1, 100%), and DHPN-induced rat tumors (7/15, 47%) [50]. Previously, we revealed the association between extracellular signal-regulated kinase 1/2 (ERK1/2) activation and mutation in *KRAS* encoding an upstream activator of ERK1/2 in NNK-induced mouse lung premalignant lesions [68].

The introduction of genetic lesions found in human lung cancer into mouse germline or pulmonary tissue has resulted in murine lung tumors that harbor similar characteristics to human lung cancer. Various transgenic models have been developed in which oncogene expression is targeted in a specific subset of lung epithelial cells (Table 2). Classic transgenic models are based on knock-in and knockout strategies [83,84,85,86], as well as ectopic expression under promotors that target specific subsets of lung epithelial cells [87,88,89,90,91,92]. Recently, inducible bitransgenic [93,94] and conditional cre/loxP models [95] have been developed that allow spatiotemporal expression of a gene in a somatic tissue to be conditionally regulated, to recapitulate its in vivo situation.

The EGFR signaling pathway plays an important role in multiple lung cancers [98]. EGFR is a transmembrane protein with cytoplasmic kinase activity that transduces important growth factor signaling from the extracellular milieu to the cell [99]. EGFR belongs to the HER/*erb*B family of receptor tyrosine kinases (RTKs), which includes HER1 (EGFR/*erb*B1), HER2 (*neu*, *erb*B2), HER3 (*erb*B3), and HER4 (*erb*B4). EGFR dimerization activates one or more downstream effectors including the MEK/ERK (MAPK kinase/ERK), PI3K/AKT (phosphatidylinositol-3-kinase/protein kinase B), STAT (signal transducer and activator of transcription), and mTOR (mammalian target of rapamycin) pathways through receptor autophosphorylation and cytoplasmic protein binding [98,99].

Many research groups have produced mouse models with similar abnormal Erbb1 (HER1) expression as seen in human lung cancers. Aberrant expression of HER1 caused lung adenocarcinoma development in doxycycline-inducible hEGFR^L858R^ (CCSP-rtTA;Tet-O_7_-hEGFR^L858R^), hEGFR^DEL^ (CCSP-rtTA;Tet-O_7_-hEGFR^DEL^), and hEGFR^VIII^ (CCSP-rtTA;TetO_7_-hEGFR^VIII^) mice [100]. Doxycycline also induced multifocal tumors in the lung parenchyma [98]. Ohashi K et al. generated transgenic mice expressing the delE748-A752 mutant version of mouse EGFR driven by the SP-C promoter, which is equivalent to the delE746-A750 mutation found in lung cancer patients [91]. This transgenic mouse model invariably developed multifocal lung adenocarcinomas of varying sizes at between 5 and 6 weeks of age. Additionally, they also generated transgenic mice expressing EGFR L858R in type II pneumocytes constitutively using the surfactant protein-C promoter [90]. This model invariably developed atypical adenomatous hyperplasia at age 4 weeks and multifocal adenocarcinoma of varying sizes at age 7 weeks. In these transgenic mice, gefitinib inhibited tumorigenesis completely [90,91].

Deletion of *EGFR* suppressed mutant *KRAS* activity and caused tumor growth temporarily. Deletion of *EGFR* also suppressed lung tumor in transgenic mice of *KRAS^G12D^, KRAS^G12D^: EGFR^ΔLep/ΔLep^, KRAS^G12D^: p53^ΔLep/ΔLep^, KRAS^G12D^: p53^ΔLep/ΔLep^: EGFR^ΔLep/ΔLep^* [101]. Lung tumors driven by strong cancer drivers (mutant *EGFR* and *Kras*) harbored few mutations in cancer-related genes, whereas tumors driven by *MYC*, a weak driver in the murine lung, harbored recurrent clonal oncogenic *KRAS* mutations [83]. Genetically engineered mice exhibit relatively simple somatic alterations compared with human cancers. Furthermore, carcinogen-induced lung carcinogenesis using transgenic mice have been reported. For example, Roh et al. [102] reported decrease in pAKT expression caused by knockout of the cell cycle-related protein, PIERCE1, in urethane- and *KRAS^G12D^*-induced lung tumor mouse model. Additionally, in the MeIQx-, urethane- and X-ray-treated models, G/C→C/G (AH), A/T→T/A (Ad), and A/T→T/A (Ad) transversions in *Kras*, respectively, were detected in a neoplasm, while *EGFR* mutations with amino acid substitution were detected in X-ray-induced tumors (4/12; 33%) [46].

Mutations in *p53* are the most common genetic alterations in human lung cancers [103]. Loss of p53 function, which is mainly induced by mis-sense mutations and rarely induced by deletions, is found in ≥75% and ~50% of small cell carcinomas and non-small cell carcinomas, respectively [100]. Ramelow et al. reported that codon 273 of *p53* (TP53-273H) is one of the most frequently mutated sites in human lung cancers and exhibited a similar oncogenic potential in lung tumors of two mice strains, A/J mice and FVB/N background mice [104]. Both strains survived more than 18 months and developed lung adenocarcinomas according to aging. Moreover, transgenic mice containing a *p53* construct with a missense mutation in exon 5 (ala135val) induced lung cancers, and 52% of the lung carcinomas contained mutations in *KRAS* (codon 61 and codon 12) [105].

Wong KK et al. reported that an increase in a single cytokine, IL-17A, without additional mutations can promote lung cancer growth by promoting inflammation using bitransgenic mice expressing a conditional *IL-17A* allele along with conditional *KrasG12D* [106]. Furthermore, pulsatile treatment with MEK inhibitors maintained T cell activity better and prolonged survival in mice with *Kras* mutant cancer [107]. Jacks T et al. revealed that lung cancer development was associated with increased bacterial burden and altered bacterial composition in the lung using *KRAS/p53* mutant mouse [108]. Barbacid M et al. revealed that combined genetic inactivation of CDK4 and RAF1 in advanced *KRAS/p53* mutant mouse lung tumors leads to effective tumor regression [109]. Transgenic animal models represent useful tools to clarify the underlying mechanisms inflammation related carcinogenesis and to test new therapeutic approaches, including immune checkpoint inhibitors, for lung cancer because they reflect the characteristics of human lung cancer rather than immunodeficiency.

In addition, the small RNAs termed microRNAs (miRNAs) regulate proliferation, morphogenesis, apoptosis, and differentiation [110]. Izzotti et al. reported that an extensive miRNA dysregulation was detected in the lungs of tobacco smoke-exposed mice [40]. Modulation of miRNA profiles was specifically related to the histopathological changes, no effect being detected in the lung fragments with non-neoplastic lung diseases (emphysema or alveolar epithelial hyperplasia), whereas a close association was observed with the presence and multiplicity of microadenomas and adenomas.

Most transgenic animal models develop lung adenocarcinoma. Few rodent models exist for testing and developing novel therapeutics in squamous cell carcinomas [111]. Furthermore, two models of murine lung neuroendocrine carcinomas induced by proneural transcription factor human achaete-scute homolog-1 (hASH-1) [112] and conditional knockdown of *p53* and *Rb* by intrabronchial Adeno-Cre infection system [113] have been reported.

## 3. Lung Carcinogenesis and Chronic Inflammation

### 3.1. Chronic Inflammation

**Figure 2:**
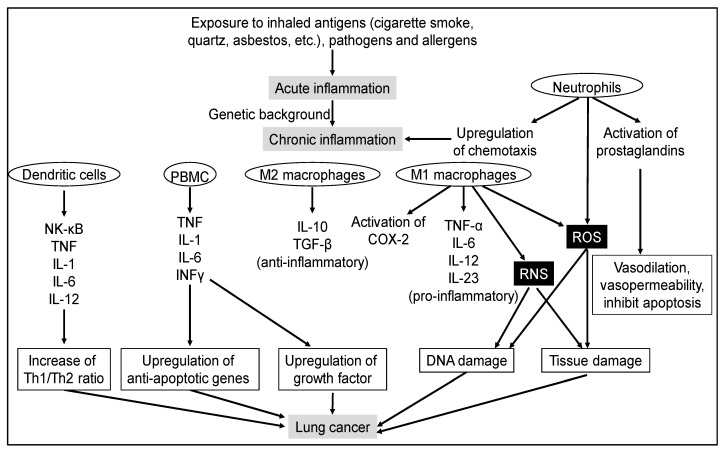
Signaling pathways of inflammatory-mediated lung carcinogenesis.

In the lungs, inflammation is commonly caused by pathogens or by exposure to toxins, pollutants, irritants, and allergens. Acute lung inflammation is dominated by neutrophils, whereas chronic reactions mainly involve macrophages and lymphocytes [114] (Figure 2). Lymphocytes are divided into two major populations: thymus-dependent T cells and bone marrow-dependent B cells. T lymphocytes have two major subsets: CD4^+^ and CD8^+^. CD4^+^ T lymphocytes, also known as helper T cells, are further subdivided into Th1 and Th2 cells that possess different cytokine profiles. Th1 cells drive cellular immunity and Th2 cells drive humoral immunity to upregulate antibody production to fight extracellular organisms. CD8^+^ T cells are mainly cytotoxic T cells. They secrete molecules that kill infected cells and tumor cells. In addition, there is a natural killer (NK) cell subset of T cells with no antigen-specific receptors [115,116]. Acute inflammation first directs neutrophil migration and then chemokine production to orchestrate formation of granulation tissue comprising cellular matrix, fibroblasts, endothelial cells, and leukocytes. Chronic inflammation occurs when resolution of acute inflammation is incomplete. In chronic lung inflammation, profibrotic and immunoregulatory Th2 cytokines (interleukin (IL)-4, IL-5, IL-9, and IL-13) are dominant [114].

Typical diseases involving chronic inflammation of the lungs include chronic obstructive pulmonary disease, interstitial pneumonia, and silicosis. Chronic obstructive pulmonary disease (COPD) is characterized by persistent airway inflammation and fixed airflow obstruction [117]. Over a third of the individuals with lung cancer have a prior diagnosis of COPD [118]. Interstitial pneumonia, a condition that causes progressive fibrosis, is a known risk factor for lung carcinogenesis independent of smoking; epidemiologically, the risk of lung carcinogenesis is 6.42 times in healthy subjects [5]. Silicosis is induced by chronic occupational exposure to quartz [7]. A significant positive relationship between cumulative silica exposure and lung cancer mortality has already been reported [119]; in this study, 1079 of 65,980 silica-exposed workers died due to lung cancer.

Immune system plays a critical role in maintaining tissue homeostasis, cell turnover, tissue remodeling, and preventing infection and cell transformation. During inflammation, numerous types of inflammatory cells are activated. Chronic lung inflammation is a significant risk factor for lung cancer. During cancer development, there are dynamic alterations in inflammatory cell population, including macrophages, neutrophils, dendritic cells, NK cells, and T and B lymphocytes [120]. Tumor-associated macrophages (TAMs) are significant for fostering tumor progression. The protumor properties of TAMs derive from regulation of angiogenic programming, production of soluble mediators that support proliferation, survival, and invasion of malignant cells, and direct and indirect suppression of cytotoxic T cell activity [121]. As neutrophils contribute directly to neoplastic transformation by amplifying the genotoxicity of urethane in lung cells via reactive oxygen species (ROS), neutrophil-released ROS can cause tissue damage that potentially favors tumorigenesis [74] (Figure 2). Neutrophils are emerging as an important player not only in tissue injury but also in post-injury tissue regeneration [122]. Several recent studies have reported conflicting functions of neutrophils in both promoting and limiting tumorigeneses [123], suggesting a context-dependent regulation [74]. Tumor-infiltrating immune cells have been widely implicated to play a significant role in carcinogenesis, through both pro- and anti-tumor effects [124]. Tumor-infiltrating T lymphocytes, particularly the CD4^+^ and CD8^+^ T cells, and their immunoregulatory cytokines, representing adaptive immunity, execute key effector cytotoxic functions in the tumor microenvironment and mediate responses to immune checkpoint inhibition [125]. B lymphocytes exist as long-lived plasma cells to produce antigen-specific antibody. Patel et al. reported that most studies largely focus on human non-small cell lung carcinoma wherein the strongest correlation between tumor-infiltrating B lymphocytes and disease-specific outcome has been shown in comparison with other forms of lung cancer [124]. These cells are considered to be key factors promoting tumor progression via their ability to release a variety of cytokines, chemokines, and cytotoxic mediators such as reactive nitrogen species (RNS), ROS, metalloproteinases, ILs, and interferons. Cancer-related inflammation affects many aspects of malignancy, including the proliferation and survival of malignant cells, angiogenesis, tumor metastasis, and tumor response to chemotherapeutic drugs and hormones [126] (Figure 2).

### 3.2. Lung Carcinogenesis and Chronic Inflammation in Rodent Models

Various animal lung tumor models with chronic inflammation have been studied (Table 3). Risk analysis of environmental chemicals on lung carcinogenesis is particularly important [35]. Many carcinogens are prevalent in the environment, and their inhalation poses a risk of carcinogenesis. Particulate matter is a major factor contributing to air quality deterioration and enters the atmosphere as a consequence of various natural and anthropogenic activities [127]. One such example is asbestos that comprises a group of naturally occurring fibrous minerals. Asbestos has been linked to a spectrum of pulmonary diseases, such as pleural fibrosis and plaques, asbestosis, benign asbestos pleural effusion, small cell lung carcinoma, non-small cell lung carcinoma, and malignant mesothelioma. Asbestos is associated with several carcinogenic mechanisms that include alterations at the chromosomal level, activation of oncogenes, loss of tumor suppressor genes, alterations in cellular signal transduction pathways, generation of reactive oxygen and nitrogen species, and direct mechanical damage to cells by asbestos fibers [128].

**Figure 3:**
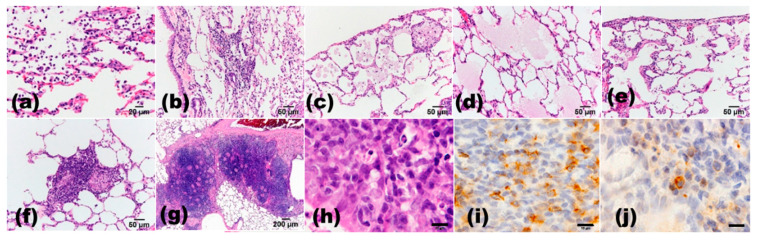
Histopathological findings in quartz (DQ-12, 2 mg/0.2 mL saline/rat i.t., after 24 weeks) -induced inflammatory changes in the rat lungs. (**a**) neutrophil infiltration in the alveolar walls and in the alveolar space; (**b**) lymphocyte infiltration in the alveolar space; (**c**) macrophage infiltration in the alveolar space; (**d**) pulmonary edema; (**e**) pulmonary fibrosis; (**f**) granuloma; (**g**) lymph follicle formation around the bronchiole; (**h**–**j**), lymphocytes infiltration around bronchiole. Scale bar, (**a**), 20 μm; (**b**–**f**), 50 μm; (**g**), 200 μm; (**h**–**j**), 10 μm. (**a**–**h**), H&E staining; (**i**), immunohistochemical staining of CD3; (**j**), immunohistochemical staining of CD20.

Quartz is classified as a Group 1 agent or human carcinogen [129] and is known to cause silicosis. Borm et al. reviewed the different modes of action of respirable quartz-induced genotoxicity in a series of independent studies [130]. They found that in vitro results of comet assay were mostly negative, except from two studies that used primary or cultured macrophages. In vivo studies confirmed the role of persistent inflammation due to quartz surface toxicity, which led to antioxidant responses in mice and rats; however, DNA damage was only observed in rats. We have developed a rat model with DHPN-initiation and quartz intratracheal instillation (i.t.) [13] as well as single quartz i.t. [7]. In our single quartz i.t. study, after 52 or 96 weeks from quartz i.t., quartz particles were observed in the lungs of all quartz-treated rats, and the quartz-treated rats had higher incidences of adenoma (85.7%) and adenocarcinoma (81.0%) than control rats (20% and 20%, respectively). In this study, the number of lung neoplastic lesions per rat positively correlated with the degree of macrophage and lymphocyte infiltration, edema, fibrosis, and lymph follicle formation around the bronchioles (Figure 3). We compared three rat strains (F344, Wistar–Hannover, and Sprague–Dawley rats) treated with the same concentration of quartz i.t. [13] and found the highest tumor-promoting effect in F344 compared with other strains, correlating to the increase in histopathological inflammatory changes (Figure 3) and IL-6 levels in the serum and bronchoalveolar lavage fluid. In another study, the Balb/c mice lung lesions induced by N-nitrosodimethylamine (NDMA) and quartz i.t. were similar to those seen in human silicosis. It was reported that silica-mediated inflammation generated an immunosuppressive microenvironment and some of the early molecular changes associated with lung carcinogenesis [131]. In addition, we found that single quartz i.t. may induce lung tumor in rats along with chronic inflammation without chemical-induced tumor promotion [7]. In our previous study, NNK-induced lung tumors in A/J mice were inflamed in the lungs by quartz i.t. (tumor-orthotopic inflammation) and sodium dextran sulfate induced inflammation in the colon (tumor and ectopic inflammation); however, strong inflammation did not persist in A/J mice, and the possibility of tumor promotion could not be confirmed [16].

In C57BL/6 mice, tumorigenesis was induced by BaP and lipopolysaccharide (LPS), a potent proinflammatory agent found in tobacco and tobacco smoke [81,132]. In an NNK plus LPS model, LPS-mediated chronic inflammation induced T-cell exhaustion, upregulated the programmed cell death-1 (PD-1)/programmed cell death ligand-1 (PD-L1) axis, and enhanced NNK-induced lung tumorigenesis through an immunosuppressive microenvironment characterized by accumulation of myeloid-derived suppressive cells and regulatory T cells [133]. Mice treated with NTCU plus LPS showed significantly increased expression of the inflammatory cytokines IL-1α, IL-6, and TNF-α [81]. Moreover, these mice exhibited significantly enhanced NF-κB, STAT3, ERK, *p*-38, and Akt activation, p53, COX-2, and Mcl-1 expression, as well as NF-κB–DNA and STAT3–DNA binding in the lungs. In recent years, heat-not-burn cigarette products have been promoted as safer and less harmful alternatives to cigarettes [134]. However, rat alveolar epithelial cells exposed to tobacco smoke extract induced oxidative stress response genes, such as *Hmox-1*, *Gsta1*, *Gsta3*, and *Nqo1* [135]. Therefore, the effects of heat-not-burn products on lung carcinogenesis need to be investigated in the future. Furthermore, BaP has an immunosuppressive role as evidenced by increased expression of *TGF-β*, *CTLA-4*, *PD-L1*, and *FOXP3* and decreased expression of *IL-12* in the lungs of BaP-treated mice as well as the increase in CD166^+^ cancer stem-like cells in the mice lungs [33]. Thus, in COPD-associated lung cancer mouse model, the immunosuppressive microenvironment could be related to tumor formation and progression [136,137].

PM2.5, a fine particle with diameter ≤ 2.5 μm [138], promotes inflammation and lung injury through IL-6/estrogen receptor (ER)β pathway. ERβ regulates IL-6 expression via MAPK/ERK and PI3K/AKT pathways [139]. Continuous exposure of PM2.5 exacerbates asthma in mouse lungs through JAK-STAT6 signaling pathway [140].

Recent studies suggest that the interaction between tobacco carcinogens and endogenous and exogenous sex steroids may be important [6]. Higher incidence of lung cancer is observed in women taking hormone replacement therapy or oral contraceptives. These results indicate that lung carcinogenesis by alterations of various inflammatory-related pathways, including the involvement of sex hormones, might influence the inflammation-related cancer progression.

### 3.3. Inhibition of Lung Cancer by Suppressing Inflammation

Various immune cells, cytokines and signaling pathways participate in inflammation-mediated lung carcinogenesis (Figure 2). Thus, various potential targets for inflammation have been proposed, including Cox-2, NK-κB, TNFα, NOS, AKT, or CXC chemokines. Furthermore, several natural compounds, such as curcumin, resveratrol, lycopene, anthocyanins, catkin, and genistein, have shown anticarcinogenic activity via the suppression of inflammation.

Administration of antibiotics and anti-inflammatory drugs (erythromycin, ampicillin, sho-saiko-to or piroxicam) was effective in suppressing the lung carcinogenesis promotion process in a DHPN-induced rat lung tumor model [78]. Furthermore, flaxseed consumption, reduced oxidative stress, reduced NNK-induced lung tumorigenesis and inflammation-mediated cytokine signaling (IL-1, 6, 8, 9, and 12α) [141].

Some recent reports have shown that lung cancer patients with COPD exhibited a higher response to immune checkpoint proteins PD-1 blockade compared with lung cancer patients without COPD [142]. Blockade of PD-L1/PD-1 immune checkpoints by monoclonal antibodies has shown measurable success in cancer therapy against a variety of tumor types, including non–small-cell lung cancer [143]. PD-L1 is expressed on both tumor cells and immune cells, and PD-1 is predominantly expressed on activated T cells. Binding of PD-L1 to PD-1 inhibits T cell effector function by inducing exhaustion and apoptosis of T cells, resulting in an immunosuppressive state [144]. Expression of PD-L1 in tumor cells plays an important role in tumor immune escape and cancer progression.

## 4. Conclusions

Many studies have clarified the molecular mechanism and roles of chronic inflammation in lung cancer. Further studies are needed to develop novel treatment strategies based on inhibition of inflammation for lung cancer. Furthermore, improvement of environmental air quality, especially with respect to reduction in tobacco smoke and dust particles, such as asbestos and silica, is a promising strategy to improve lung cancer motility and mortality.

## Figures and Tables

**Table 1:** Chemical induced lung tumorigenesis in animal models.

Chemical	Dose	Animals	Histopathological Type	Reference
Tobacco related				
NNK	2 mg/0.1 mL saline/mouse, single i.p.	A/J mice	AH, AD, AC	[16,21,35,44,45,46,50,62,63,64,65,66,67,68]
	2 mg/0.1 mL saline/mouse, 2 times i.p.	A/J mice	AH, AD, AC	[22,44,69,70,71]
	10 mg/mL saline/rat, 3 times i.p.	F344 rats	AH	[17,49]
	20 mg/mL saline/rat, single i.p.	F344 rats	AH	[49]
	50 mg/kg BW, i.p.	A/J mice	Tumor	[72]
Urethane	1 g/kg BW, single i.p.	FVB mice	AAH, AD	[14]
	1 mg/kg BW, 10 times i.p.,	C57/BL6 mice	AAH, AD, AC	[73]
	250 mg/kg BW, i.p.	A/J mice	AD	[46]
	1 mg/kg BW, 10 times i.p.	F344 rats	AH	[19,21]
	5 mg/mouse, single i.p.	A/J mice	AH	[21]
	1 mg/g BW, single i.p.	FVB/J mice	AD	[74]
DEN	15 μg/g BW, single i.p.	FVB/N mice	AC	[29]
	15 μg/g BW, single i.p.	A/J mice	AC	[75]
	100 mg/kg BW, single s.c.	Syrian golden hamster	BH, squamous papilloma	[38]
MeIQx	600 ppm MeIQx in a basal diet for 12 weeks	A/J mice	AH, AD	[46,50]
	0.01, 0.1, 1, 10, 100 ppm MeIQx in a basal diet for 32 weeks	A/J mice	AH, AD	[67]
BaP	20 mg/kg BW, i.t.	F344 rats	AH	[19,21]
Tobacco smoke	expose MCS for 22 weeks	FVB/N mice	AD, AC	[39]
	whole-body to MCS during the first 4 months of life	H neonatal mice	AH, AD, AC	[40]
	expose MCS for 12 weeks	Syrian golden hamster	BH, squamous papilloma	[38]
Others				
DHPN	100 or 500 ppm, drinking water	Wistar rats	AD, AC, SCC, ADSC	[76]
	2000 ppm, drinking water, 12 weeks	Wistar rats	AC, SCC, ADSC	[77,78]
	0.1% drinking water, 2 weeks	F344 rats	AH, AD, AC	[17,19,20,21,22,49,50,51]
	0.2% drinking water, 1 week	F344 rats	AH, AD, AC	[79]
Aflatoxin B_1_	150 mg/kg BW, i.p. divided into 24 doses over 8 weeks	AC3F1 mice	AH, AD, AC	[54]
Aflatoxin G_1_	100 μg/kg BW, orally administered	Balb/c mice	AD	[55]
4-NQO	2 mg/mL, s.c.	ICR mice	AD	[57]
	10 mg/kg BW, single s.c.	TSOD mice	AH, AD, AC	[56]
DMN	30 mg/kg BW, single i.p.	F344 rats	AH, AD	[19,21]
X-ray	3.0 Gy X-rays	Wistar rats	AD, AC	[46]
NTCU	skin painted, 2 times/week, for 35–40 weeks	Swiss mice	SCC, ADSC, AC, a single ciliated-cell tumor	[59]
	skin painted, 2 times/week, for 8 months	Swiss mice	SCC	[80]
	0.5 mmol/L/mouse, intranasal administration, once a week for 26 weeks	A/J mice	BH, SCC	[81]

NNK, 4-(methylnitrosamino)-1-(3-pyridyl)-1-butanone; DEN, diethlnitrosamine; MeIQx, 2-amino-3,8 dimethylimidazo[4, 5-f]quinoxaline; DHPN, N-bis(2-hydroxypropyl)nitrosamine; BaP, Benzo[a]pyrene; MCS, mainstream cigarette smoke; 4NQO, 4-Nitroquinoline 1-oxide; DMN, N-nitrosodimethylamine; N-nitroso-tris-chloroethylurea, NTCU; i.p., intraperitoneal injection; i.t., intratracheal instillation; s.c., subcutaneous; AH, alveolar hyperplasia; AAH, atypical adenomatous hyperplasia; AD, adenoma; AC, adenocarcinoma; SCC, squamous cell carcinoma; ADSC, adenosquamous carcinoma; BH, bronchial hyperplasia; BW, body weight.

**Table 2:** Gene Mutation and Transgenic Animal Models [84].

Methods	Genes
Knockout	*Fhit-Vhl* [85]
Knock-in	*K-ras*G12D [83,86]
Promotors	
keratin	papillomavirus-16 E6/E7 [87]
surfactant protein C	simian virus large T antigen [88], *c-myc,* epidermal growth factor [89], *Ron, Raf-1, p53* [90,91]
Clara cell secretory protein (CCSP)	simian virus large T antigen [92]
calcitonin gene-related peptide	
Reverse tetracycline transactivator inducible system	CCSP-rtTA/Tet-Op-FGF7 [93]
	CCSP-rtTA/Tet-Op-K-*ras*4G12D [94]
Conditional cre/loxP	Lox-STOP-Lox-K-*ras*G12D [95]
Compound conditional models	conditional activation of K*ras*2 with conditional inactivation of *Rb* or *p53* by cre/loxP [96]
	conditional knock-in of LSL-K-*ras* G12D and LSL-*p53*R270H [97]

**Table 3:** Lung carcinogenesis models with inflammation.

Lung Tumor Inducer	Inflammatory or Anti-Inflammatory Substances	Animals	Histopathological Type of Tumors	References
DHPN	quartz and dextran sulfate sodium	F344 rats	AD, AC	[13]
	erythromycin, ampicillin, sho-saiko-to or piroxicam	Wistar rats	AC, SCC, ADSC	[78]
	None	Wistar rats	AC, SCC, ADSC	[78]
NNK	quartz	A/J mice	AD, AC	[16]
	LPS	FVB/NJ mice	AD, AC	[133,136]
	flaxseed	A/J mice	AD, AC	[141]
NDMA	quartz	Balb/c mice	AD, AC	[131]
BaP	LPS	C57BL/6	AD, AC	[132]
NTCU	LPS	A/J mice	SCC	[81]
None	quartz	F344 rats	AD, AC	[7]

NDMA, N-nitrosodimethylamine; LPS, lipopolysaccharide; AD, adenoma; ADC, adenocarcinoma; ADSC, adenosquamous carcinoma, SCC, squamous cell carcinoma; None, no use of tumor inducer or inflammatory substance.
